# High-Quality Whole-Genome Sequence of an Estradiol-Degrading Strain, Novosphingobium tardaugens NBRC 16725

**DOI:** 10.1128/MRA.01715-18

**Published:** 2019-03-14

**Authors:** J. Ibero, D. Sanz, B. Galán, E. Díaz, J. L. García

**Affiliations:** a Department of Microbial and Plant Biotechnology, Centro de Investigaciones Biológicas, Consejo Superior de Investigaciones Científicas, Madrid, Spain; b Department of Applied Biotechnology, Institute for Integrative Systems Biology (I2SysBio), Universidad de Valencia-CSIC, Valencia, Spain; Indiana University, Bloomington

## Abstract

In this work we report the complete sequence and assembly of the estradiol-degrading bacterium Novosphingobium tardaugens NBRC 16725 genome into a single contig using the Pacific Biosciences RS II system.

## ANNOUNCEMENT

Estrogens are C18 steroid hormones that play several roles in animal physiology (1) but also are important endocrine-disrupting compounds (EDCs) affecting aquatic fauna, even at extremely low concentrations (2). The occurrence and abundance of estrogens in aquatic environments present a serious risk to public health (3), and they have been classified by the World Health Organization as group 1 carcinogens (https://monographs.iarc.fr/list-of-classifications-volumes/).

Although the presence and removal of estrogens via biodegradation have been studied to some extent (4), current knowledge of microbial estrogen degradation pathways is still very limited (5). The bacterial degradation of estrogens is an uncommon trait, and only a few bacteria are known to completely metabolize them (6 – 8).

The alphaproteobacterium Novosphingobium tardaugens NBRC 16725 (described as strain ARI-1) was isolated at a sewage treatment plant in Tokyo, Japan, and is able to use estradiol, estrone, and estriol as its only carbon source (9, 10). It is a Gram-negative, aerobic, rod-shaped, and nonmotile bacterium. The genome of this strain was previously sequenced using 454 GS-FLX Titanium and Illumina HiSeq 1000 technologies and assembled in 54 contigs (contig *N*_50_, 486,386 bp; contig *L*_50_, 4 bp) (BioProject number PRJDB314).

Here, we report the complete sequence and assembly of the N. tardaugens NBRC 16725 genome into a single contig using the Pacific Biosciences (PacBio) RS II technology that offers very long and unbiased reads that are uniquely suited for closing genome assemblies.

**FIG 1:**
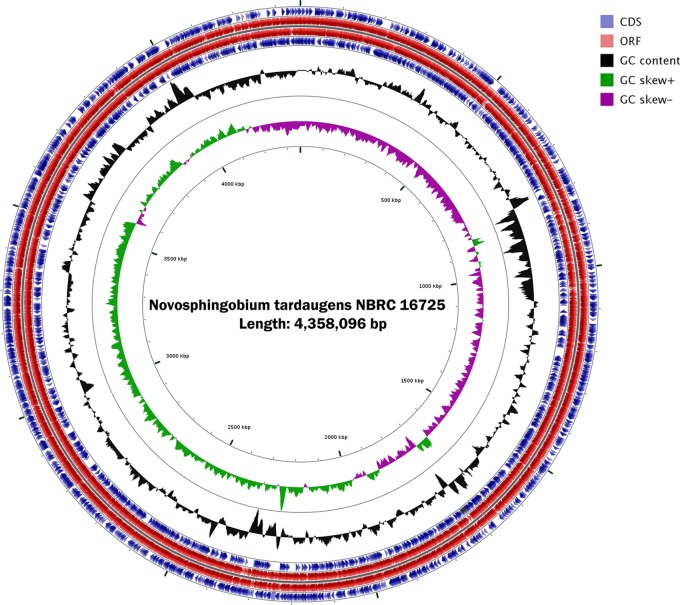
Graphical map of the circular genome of Novosphingobium tardaugens NBRC 16725 obtained using the GCView Server (21) comparative genomic tool. Colors show CDS (blue), ORFs (red), GC content (black), GC skew + (green) and GC skew – (purple).

N. tardaugens NBRC 16725 was purchased from the Leibniz Institute DSMZ—German Collection of Microorganisms and Cell Cultures. The strain was grown at 30°C in nutrient broth medium in an orbital shaker. Genomic DNA was isolated using the phenol chloroform extraction method (11). The G-tube method was used to construct a 10-kb SMRTbell template library of 9,172 kb, which was checked on an Agilent Technologies 2100 Bioanalyzer instrument. The sequencing reagent used was single-molecule real-time (SMRT) cell 8Pac v3 with the DNA polymerase binding kit P6. Sequencing yielded 1,248,591,484 total bases and 205,778 total reads with a mean subread length of 6,067 bp and an *N*_50_ value of 7,616 bp. All raw data were deposited in the SRA database under accession number SRR8271516. The PacBio reads were assembled *de novo* using RS Hierarchical Genome Assembly Process (HGAP) v3.0 software (12) with default options, yielding a single contig with a total length of 4,358,096 bp and a 61.2% GC content (Fig. 1). Pairwise alignment between that single contig sequence and a previous assembly using Geneious v11.0.5 software revealed 99.8% base pair identity, and 175,514 additional nucleotides were sequenced that were distributed in 26 gaps existing between previously available contigs. The whole assembled nucleotide sequence was deposited in GenBank under the accession number CP034179.

Protein-, rRNA-, and tRNA-coding genes were annotated using the NCBI Prokaryotic Genome Automatic Annotation Pipeline (PGAAP) (13) and Rapid Annotations using Subsystems Technology (RAST) (14). This genome contained 4,097 open reading frames (ORFs) with 4,040 coding DNA sequences (CDS), 48 tRNA-coding sequences, and 6 rRNA-coding sequences.

The N. tardaugens NBRC 16725 genome (4.36 Mbp) was found to be highly similar (70.79%) to that of Novosphingobium subterraneum strain DSM 12447 (15) using average nucleotide identity based on BLAST (ANIb) analysis (16).

The *oecA*, *oecB*, and *oecC* genes involved in estradiol degradation (17) are located in two distinct gene clusters as in *Sphingomonas* sp. strain KC8 (7), covering a 76-kb region that also contains the testosterone degradation cluster (18), which was segregated in the previous assembly. The genome includes a third cluster containing the *sal2*, *scd2AB*, and *stdA2* genes involved in cholic acid degradation (19, 20).

### Data availability.

This genome sequencing project has been deposited in GenBank under the accession number PRJNA505257. The version described in this paper is version CP034179. All raw data have been deposited in the SRA database under accession number SRR8271516.
